# Molecular epidemiology and antimicrobial resistance of group a streptococcus recovered from patients in Beijing, China

**DOI:** 10.1186/s12879-020-05241-x

**Published:** 2020-07-13

**Authors:** Hongxin Li, Lin Zhou, Yong Zhao, Lijuan Ma, Xiaoyan Liu, Jin Hu

**Affiliations:** 1 Department of Dermatology, Children’s Hospital Affiliated to Capital Institute of Pediatrics, Beijing, 100020 China; 2 Department of Clinical Laboratory, Children’s Hospital Affiliated to Capital Institute of Pediatrics, Beijing, 100020 China; 3 The Sixth Medical Centre of PLA, General Hospital, Beijing, 100048 China

**Keywords:** Group a streptococcus, GAS, Streptococcal M protein, *emm* types, Superantigens, Antimicrobial resistance, Scarlet fever, Children

## Abstract

**Background:**

Group A streptococcus (GAS) is an important human pathogen responsible for a broad range of infections. Epidemiological surveillance has been crucial to detect changes in the geographical and temporal variation of the disease pattern. The objective of this study was to investigate the molecular epidemiological characteristics and antimicrobial resistance of GAS isolates from patients in Children’s Hospital in Beijing.

**Methods:**

From 2016 to 2017, pharyngeal swab samples were collected from the outpatients in Children’s Hospital, Capital Institute of Pediatrics, who were diagnosed with scarlet fever. Antimicrobial susceptibility test was performed according to the distribution of conventional antibiotics and Clinical and Laboratory Standards Institute (CLSI) recommendations. The distribution of the macrolide-resistance genes (*ermB, ermA, mefA*), *emm* (M protein-coding gene) typing, and superantigens (SAg) gene profiling were examined by polymerase chain reaction (PCR).

**Results:**

A total of 297 GAS isolates were collected. The susceptibility of the isolates to penicillin, ceftriaxone, and levofloxacin was 100%. The resistance rate to erythromycin and clindamycin was 98.3 and 96.6%, respectively. The dominant *emm* types were *emm12* (65.32%), *emm1* (27.61%), *emm75* (2.69%), and *emm89* (1.35%). Of the 297 isolates, 290 (97.64%) carried the *ermB* gene, and 5 (1.68%) carried the *mefA* gene, while none carried the *ermA* gene. The most common superantigen genes identified from GAS isolates were *smeZ* (96.97%)*, speC* (92.59%), *speG* (91.58%), *ssa* (85.52%), *speI* (54.55%), *speH* (52.19%), and *speA* (34.34%). Isolates with the genotype *emm1* possessed *speA*, *speC*, *speG*, *speJ*, *speM*, *ssa*, and *smeZ*, while *emm12* possessed *speC*, *speG*, *speH*, *speI*, *speM*, *ssa*, and *smeZ* superantigens.

**Conclusions:**

The prevalent strain of GAS isolates in Beijing has a high resistance rate to macrolides; however, penicillin can still be the preferred antibiotic for treatment. Erythromycin resistance was predominantly mediated by *ermB.* The common *emm* types were *emm12* and *emm1.* There was a correlation between *emm* and the superantigen gene. Thus, long-term monitoring and investigation of the *emm* types and superantigen genes of GAS prevalence are imperative.

## Background

*Streptococcus pyogenes* (Lancefield group A streptococcus; GAS) is a major pathogen causing infectious diseases in children. It causes suppurative and non-suppurative diseases, such as erysipelas, suppurative tonsillitis, scarlet fever, rheumatic fever, and glomerulonephritis [1]. Globally, there are about 616 million cases of GAS pharyngitis every year, among which, 17,800 cases are new infections, and about 517,000 patients with severe GAS are deceased every year [2]. Recently, the positive rate of GAS was estimated at 21.2% in the pharyngeal culture of patients diagnosed as “streptococcal infection/tonsillitis/angina” [3]. Moreover, the incidence of streptococcal pharyngitis is common in children aged 0–14 years. From 2012 to 2014, the average number of positive cases of streptococcal culture was 2685.1/100,000 children in Beijing, including 1652.7 outpatient visits [4]. In 2011, scarlet fever broke out in mainland China and Hong Kong, with a sharp increase in incidence [5, 6]. Penicillin is the preferred clinical treatment of GAS infection, while erythromycin is the first alternative antibiotic for patients allergic to penicillin, followed by clindamycin. The resistance rate to macrolidesis gradually increasing, which might be related to the overuse of such antibiotics [7, 8]. In our previous study, 95 isolates were recovered from suppurative tonsillitis, acute glomerulonephritis, scarlet fever and streptococcal dermatitis. The resistance rates of the isolates to erythromycin, clindamycin and tetracycline were 98.9, 100, 94.7% respectively [9].

The M protein encoded by the *emm* gene is the main pathogenic factor of GAS, and different types vary in pathogenicity. Therefore, *emm* typing is employed to track outbreak and routinely monitor GAS diseases. From 2011 to 2013, the proportion of *emm12.*0 in children with GAS infection in Xicheng District of Beijing decreased gradually, and *emm1.0* increased every year [10]. The interplay among GAS diseases, *emm* types, superantigen gene, and antimicrobial resistance needs further investigation [11, 12]. The resistance of GAS to macrolides is related to the mechanism underlying target modification mediated by *ermA* and *ermB* and the pumping mechanism mediated by *mefA*. Intriguingly, different primary mechanisms of resistance regulate various epidemic strains [13]. The superantigen genes are the main virulence factors and closely related to the pathogenicity of GAS. Hitherto, 11 superantigen genes, including *speA*, *speC*, *speG*, *speH*, *speI*, *speJ*, *speK*, *speL*, *speM*, *smeZ*, and *ssa*, were found to be distributed among various strains [14, 15]. Previously, the *emm* typing and superantigen distribution of GAS strains from different regions of mainland China were investigated, which indicated that the typing and antimicrobial resistance were slightly different [16, 17]. In this study, we recovered GAS isolates from patients with scarlet fever in Children’s Hospital, Capital Institute of Pediatrics from 2016 to 2017, and conducted antimicrobial susceptibility test, *emm* genotype analysis, and combined analysis of superantigen to assess the molecular epidemiological characteristics and antimicrobial resistance mechanisms of GAS strains.

## Methods

### Strain collection

A total of 297 cases of GAS isolates were recovered from pediatric patients presenting scarlet fever in the Children’s Hospital, Capital Institute of Pediatrics, from January 2016 to December 2017. Throat swabs were obtained from patients by two physicians for routine microbiologic analysis.

### Bacterial identification

The throat swab samples were inoculated on a Columbia blood plate (BD, USA) and cultured in a CO_2_ incubator at 37 °C for 24–36 h. A single round colony with the transparent hemolytic ring was selected, cultured, and evaluated by Gram staining. The Streptococcus Grouping Kit (Oxoid, Basingstoke, UK) was used to classify the suspicious colonies.

### Antimicrobial susceptibility testing

According to the distribution of conventional antibiotics and recommendations of Clinical and Laboratory Standards Institute (CLSI), disk diffusion method (K-B method) was used to test the susceptibility of the isolated *Streptococcus pyogenes* to ten antibiotics. The distance between each disk was > 24 mm, and the distance between the center of the disk and edge of the dish was > 15 mm. The plate was incubated at 37 °C for 18–24 h and only two plates were stacked. The susceptibility of bacteria was determined by the diameter of the inhibition growth and CLSI standard [18]. *Streptococcus pneumoniae* ATCC 49619 was used as a control strain..

### DNA extraction of GAS genome

DNA extraction of GAS genome was performed according to the recommended method by the Center for Disease Control and Prevention (CDC; https://www.cdc.gov/streplab/groupa-strep/emm-typing-protocol.html), one ring of GAS was suspended in 300 μL saline and incubated at 70 °C for 15 min. The precipitate, obtained by centrifugation and suspended in 50 μL TE (pH 8.0), 10 μL mutanolysin (3000 U/mL), and 2 μL hyaluronidase (30 mg/mL), was reheated at 100 °C for 10 min and centrifuged to obtain genomic DNA.

### *emm* genotyping

The *emm*-typing of all isolates was performed according to the protocols and recommendations of the CDC. The sequence data were uploaded to the *emm* typing database (https://www2.cdc.gov/vaccines/biotech/strepblast.asp) for comparison. A sequence was considered to belong to a specific *emm* gene when the first 160 nt of the sequence exhibited >95% sequence identity with that of the reference *emm* gene.

### Erythromycin-resistance gene detection

All GAS isolates were screened for the presence of *ermB*, *ermA*, and *mefA* as previously reported [19] Primer sequences are listed in Table 1. The reaction system (25 μL) contained 1 μL 2 mM DNA template, 1 μL (10 mM) for each primer, 0.2 μL Taq DNA polymerase (5 U/μL), 2 μL of 2.5 mM dNTPs, 2.5 μL of 10× Taq buffer (2.5 mM MgCl_2_ plus; Takara Biotechnology Co.), and 17.3 μL water. The initial denaturation was performed for 1 min at 94 °C, followed by denaturation for 30 s at 94 °C annealing for 30 s at 54 °C, extended for 30 s at 72 °C for 29 cycles and final extension for 3 min at 72 °C.

**Table 1:** Primer sequences for erythromycin-resistance genes

Gene	Primer direction	Primer sequence	Amplicon size (bp)
*ermB*	Forward	5′-ATTGGAACAGGTAAAGGGC-3′	639
	Reverse	5′-GAACATCTGTGGTATGGCG-3′	
*ermA*	Forward	5′-AACTTGTGGAAATGAGTCAACGG-3′	530
	Reverse	5′-CAGAATCTACATTAGGCTTAGGG-3′	
*mefA*	Forward	5′-AGTATCATTAATCACTAGTGC-3′	348
	Reverse	5′-TTCTTCTGGTACTAAAAGTGG-3	

### Superantigen detection

Eleven superantigen genes (*speA*, *speC*, *speG*, *speH*, *speI*, *speJ*, *speK*, *speL*, *speM*, *ssa*, and *smeZ*) were amplified by PCR using the genomic DNA extracted during *emm* typing as previously described [20]. By comparing the size of the products with the predicted positive fragments, the expression of superantigen in GAS was determined. The primers used for amplification are listed in Table 2.

**Table 2:** Primers for PCR of virulence and superantigen genes

Gene	Primer direction	Primer sequence	Annealing Temperature (°C)	Amplicon Size (bp)
*speA*	Forward	5′-ATGGAAAACAATAAAAAAGTATTG-3’	52	756
	Reverse	5′-TTACTTGGTTGTTAGGTAGACTTC-3’		
*spec*	Forward	5′-AATTTCGATTCTGCCGCTTA-3’	52	400
	Reverse	5′-GCAGGGTAAATTTTTCAACGAC-3’		
*speG*	Forward	5′-TCATGTGTTTTTAGCTATGGAAGTC-3’	52	590
	Reverse	5′- ACTGTCTCGACTTAAAAGCTTATCA-3’		
*speH*	Forward	5′-AGATTGGATATCACAGG-3’	52	416
	Reverse	5′- CTATTCTCTCGTTATTGG-3’		
*speI*	Forward	5′- AATGAAGGTCCGCCATTTTC-3’	52	516
	Reverse	5′-TCTCTCTGTCACCATGTCCTG-3’		
*speJ*	Forward	5′-GATAGTGAAAATATTAAAGACG-3’	52	630
	Reverse	5′- TTATTTAGTCCAAAGGTAAATATC-3’		
*speK*	Forward	5′-GTGTGTCTAATGCCACCGTCT-3’	52	564
	Reverse	5′-GGAACATATATGCTCCTAGAT-3’		
*speL*	Forward	5′-CAGCACCTTCCTCTTTCTCG-3’	52	459
	Reverse	5′-GGAAAAAGAGGGACGCAAG-3’		
*speM*	Forward	5′-GGATGAGTGAATAAATCGGTAAAC-3’	55	425
	Reverse	5′-AGTCTGGGACGATGATAA-3’		
*ssa*	Forward	5′-TGATCAAATATTGCTCCAGGTG-3’	52	502
	Reverse	5′-TCCACAGGTCAGCTTTTACAG-3’		
*smeZ*	Forward	5′-CTTCAATATTCATTGCAATAATTTC-3’	52	430
	Reverse	5′-TGTAACTGTTGTTTTGTTAGTTGAT-3’		

## Results

### Patients’ characteristics

297 isolates were recovered from 1183 throat swab samples. For the 297 patients, the mean age was 6.17 years, the median was 6 years (range from 1 to 13 years), and 175 were boys, 122 were girls.

### Antimicrobial susceptibility testing results

All 297 GAS isolates were sensitive to penicillin, ceftriaxone, cefotaxime, cefepime, vancomycin, and levofloxacin. They were resistant to erythromycin, clindamycin and tetracycline with the resistance rate 98.3%(292/297), 96.6% (287/297) and 90.23% (268/297), respectively (Table 3).

**Table 3:** Antimicrobial susceptibility test of 297 isolates of GAS from Children’s Hospital during 2016 to 2017

Antibiotic	Susceptibility (n/%)		
	Susceptible	Intermediate	Resistant
Penicillin C	297/100	0	0
Ceftriaxone	297/100	0	0
Clindamycin	8/2.69	2/0.67	287/96.6
Erythromycin	3/1.01	2/0.67	292/98.3
Tetracycline	16/5.39	13/4.38	268/90.23
Vancomycin	297/100	0	0
Chloramphenicol	283/95.29	12/4.04	2/0.67
cefepime	297/100	0	0
cefotaxime	297/100	0	0
Levofloxacin	297/100	0	0

### Distribution of *emm* types

Overall, 9 *emm* types were detected in GAS isolates, including 28 subtypes from 2016 to 2017. The majority of the cases were *emm12* (65.32%, 194/297), *emm1* (27.61%, 82/297), *emm75* (2.69%, 8/297), and *emm89* (1.35%, 4/297). *emm12.0* and *emm1.0* were the most prevalent subtypes, accounted for 46.8 and 26.26%, respectively. A variant subtype (*stg485.0*) was also detected. The distribution of the *emm* types is shown in Table 4.

**Table 4:** Distribution of the *emm* genotypes of GAS isolates from Children’s Hospital during 2016 to 2017

*emm* types	*emm* subtypes	Count (n)
*emm1*	*emm1.0*	78
	*emm1.25*	1
	*emm1.3*	1
	*emm1.33*	2
*emm12*	*emm12.0*	139
	*emm12.12*	1
	*emm12.13*	1
	*emm12.19*	27
	*emm12.20*	1
	*emm12.21*	2
	*emm12.30*	1
	*emm12.36*	4
	*emm12.37*	8
	*emm12.40*	2
	*emm12.66*	2
	*emm12.69*	2
	*emm12.70*	1
	*emm12.72*	2
	*emm12.76*	1
*emm6*	*emm6.19*	1
	*emm6.4*	1
	*emm6.89*	1
*emm75*	*emm75.0*	8
*emm89*	*emm89.0*	4
*stg485*	*stg485.0*	1
*emm225*	*emm225*	1
*emm3*	*emm3.1*	3
*emm4*	*emm4.0*	1
Total	28	297

### *emm* types and erythromycin-resistance genes

Among the 297 isolated GAS strains, 290 (97.64%) carried *ermB*, while 5 (1.68%) carried *mef*A and none carried *ermA* (Table 5). Three erythromycin sensitive strains were found among the isolates, distributed in subtype *emm12.0* and *emm3.1.* None of the three isolates showed the presence of *ermA*, *ermB*, and *mefA*. Clindamycin-sensitive strains were distributed in *emm12.0* and *emm3.1* subtypes. The positive rates of *ermB, ermA, and mefA* in *emm12* and *emm1* strains were 45.79, 0, 0.34 and 26.3%, 0, 0.67%, respectively.

**Table 5:** Distribution of *emm* types erythromycin-resistant genes and superantigens in 297 GAS isolates from Children’s Hospital during 2016 to 2017

*emm* genes		erythromycin-resistant genes			superantigens										
Type	n.	*ermB* (n/%)	*ermA* (n/%)	*mefA* (n/%)	*speA* (n)	*speC* (n)	*speG* (n)	*speH* (n)	*speI* (n)	*speJ* (n)	*speK* (n)	*speL* (n)	*speM* (n)	*ssa* (n)	*smeZ* (n)
*emm1.0*	78	78/26.3	0/0	2/0.67	70	76	74	3	5	55	1	2	13	66	76
*emm12.0*	139	136/45.79	0/0	1/0.34	11	125	126	123	124	6	0	3	40	130	134
*emm3.1*	3	1/0.34	0/0	1/0.34	2	2	3	0	1	0	3	0	1	1	3
*emm4.0*	1	1/0.34	0/0	0/0	0	1	1	0	0	0	0	0	0	1	1
*emm6.19*	1	1/0.34	0/0	0/0	1	1	1	0	0	0	1	0	1	0	1
*emm6.4*	1	1/0.34	0/0	0/0	1	1	1	0	0	0	1	0	1	0	1
*emm6.89*	1	1/0.34	0/0	0/0	1	1	1	0	0	0	0	0	0	0	1
*emm75.0*	8	8/2.69	0/0	0/0	1	8	7	6	8	1	0	8	6	0	8
*emm89.0*	4	4/1.35	0/0	0/0	0	4	4	0	0	0	0	0	1	0	4
*emm225*	1	1/0.34	0/0	0/0	1	1	1	0	0	0	0	0	0	1	1
*stg485.0*	1	1/0.34	0/0	0/0	0	0	0	0	0	0	0	0	0	0	0
*emm1.25*	1	1/0.34	0/0	0/0	1	1	1	0	0	1	0	0	0	1	1
*emm1.3*	1	1/0.34	0/0	0/0	1	1	1	0	0	1	0	0	0	1	1
*emm1.33*	2	2/0.67	0/0	0/0	2	2	2	0	0	2	0	0	0	2	2
*emm12.12*	1	1/0.34	0/0	0/0	0	1	1	1	1	0	0	0	0	1	1
*emm12.13*	1	1/0.34	0/0	0/0	0	0	1	1	1	0	0	1	1	1	1
*emm12.19*	27	27/9.09	0/0	0/0	4	26	25	3	3	0	0	1	4	27	27
*emm12.20*	1	1/0.34	0/0	1/0.34	1	1	1	1	1	0	0	0	0	1	1
*emm12.21*	2	2/0.67	0/0	0/0	0	1	1	1	2	0	0	0	0	1	1
*emm12.30*	1	1/0.34	0/0	0/0	0	0	1	1	1	0	0	0	1	0	1
*emm12.36*	4	4/1.35	0/0	0/0	2	4	4	3	3	0	0	0	0	4	4
*emm12.37*	8	6/2.02	0/0	0/0	2	8	7	7	7	0	0	0	2	8	8
*emm12.40*	2	2/0.67	0/0	0/0	0	2	1	2	2	0	0	0	1	2	2
*emm12.66*	2	2/0.67	0/0	0/0	0	2	2	2	2	0	0	0	1	2	2
*emm12.69*	2	2/0.67	0/0	0/0	1	2	1	0	0	0	0	0	0	2	2
*emm12.7*	1	1/0.34	0/0	0/0	0	1	1	0	0	0	0	0	0	0	1
*emm12.72*	2	2/0.67	0/0	0/0	0	2	2	0	0	0	0	0	0	2	2
*emm12.76*	1	1/0.34	0/0	0/0	0	1	1	1	1	0	0	0	0	0	1
Percentage(%)		97.64	0	1.68	34.34	92.59	91.58	52.19	54.55	22.22	2.02	5.05	24.58	85.52	96.97

### *emm* type and superantigen distribution

Of 297 isolates, the most common superantigen genes identified from *S. pyogenes* were *smeZ* (96.97%)*, speC* (92.59%), *speG* (91.58%), and *ssa* (85.52%), while the expression rate of other superantigens was slightly lower: *speI* (54.55%), *speH* (52.19%), *speA* (34.34%), *speM* (24.57%), *speJ* (22.22%), *speL* (5.05%), and *speK* (2.02%). *emm1* tended to harbor *speA*, *speC*, *speG*, *speJ*, *speM*, *ssa*, and *smeZ*, but less *speI*, *speK*, and *speL*. *emm12* tended to harbor *speC*, *speG*, *speH*, *speI*, *speM*, *ssa*, and *smeZ*, with little or no *speJ*, *speK*, *speL*. Variant *stg485* did not express any detected superantigens. The details of superantigen distribution are shown in Table 5.

## Discussion

*S. pyogenes* or GAS is a leading pathogen causing infectious diseases in children. The GAS infection manifests as mild non-invasive diseases, such as acute pharyngitis or life-threatening invasive diseases, such as sepsis and toxic shock syndrome [15]. Scarlet fever is a acute infectious disease caused by GAS, that can affect people of all ages, but it is most often seen in children. Before the advent of antibiotics, scarlet fever was extremely serious, often causing long periods of illness, many dangerous complications, and even death. A re-emergence of scarlet fever has been noted in Hong Kong, mainland China, South Korea, and England, UK, and other countries around the world since 2008 to 2014 [21–23]. Penicillin has always been the preferred treatment for the GAS infection. In penicillin allergic patients, macrolides are the most commonly used antibiotics for treating streptococcal infections. However, the resistance rate of macrolides has also been increasing gradually [7]. Of the GAS isolates recovered from the throat swabs of children with pharyngitis in Madison, Wisconsin, 15% demonstrated nonsusceptibility for clindamycin and erythromycin, and inducible resistance (positive D-test) was detected in 12% isolates [24]. *S. pyogenes* isolates collected from infected patients from 7 cities/provinces in China during the years 2009–2016, were phenotypically susceptible to penicillin, ampicillin, cefotaxime, and vancomycin, whereas 93.5, 94.2, and 86.4% were resistant to erythromycin, clindamycin, and tetracycline, respectively [25]. In this study, the GAS isolates recovered from children with scarlet fever were highly sensitive to penicillin, cephalosporin, levofloxacin, and vancomycin while the resistance rates to erythromycin, clindamycin and tetracycline are 98.3, 96.6 and 90.23%, respectively (see Table 3). No significant shift was detected in the resistance rate of GAS isolates to antibiotics between 2016 and 2017. These findings were consistent with those from previous study in 2013 that the resistance rates of isolates obtained from scarlet fever in Beijing to erythromycin, clindamycin and tetracycline were 99.3, 99.3 and 88.2% respectively [26]. However, Erythromycin resistance was found in 51.4% of isolates in India [27]. In Brazil, resistance to erythromycin and clindamycin was 15.4% [28]. Thus, antimicrobial susceptibility test is suggested before choosing erythromycin as an alternative treatment for penicillin-allergic patients.

Traditionally, GAS infection patients with penicillin allergy are commonly treated with macrolide antibiotics. In the late 1990s, the resistance rate of GAS isolates to erythromycin in most regions of China was less than 50%. Around 2008, the resistance rate of GAS to erythromycin was 95–100%, while that for the isolates in Taiwan decreased from 53.1% in 2000 to 0% in 2010, but rapidly increased to 65% in 2011. The genes involved in erythromycin resistance were *mefA* (53.1%), *ermB* (35.9%), and *ermTR* (10.9%) [29]. In this study, the resistance rate to erythromycin was 98.3%, that was much higher than that detected in North America and some European countries (9.6–35.8%). Of the total 297 isolates, 290 (97.64%) harbored the *ermB* gene, 5 (1.68%)harbored *mefA*, none harbored *ermA*. This phenomenon differed from that in the USA, Italy, Chile, and Canada where erythromycin-resistant strains of GAS are mainly M-resistant phenotypes mediated by *mefA*. The target modification mechanism mediated by *ermB* is the main resistance mechanism of GAS in China. The pattern of antibiotic resistance fluctuates worldwide. In a study in India, 51.4% of the GAS isolates were resistant to erythromycin, of which, 65.1% harbored *ermB* and 32.5% harbored *mef*A as the only genes resistant to macrolides, while 2.2% harbored both *ermB* and *mefA* [8]. The resistance rate of erythromycin and clindamycin in Korea decreased from 51.0 and 33.7% in 2002 to 9.8 and 8.8% in 2004, respectively. The sharp decline in erythromycin resistance in a short period may be related to the change in *emm* type distribution in the community [30]. In Portugal, the resistance rates of erythromycin and clindamycin were 14% (carrying the *erm*B gene) and 9% (harboring the *ermTR* gene) in 2010–2015, respectively [31]. Thus, it could be deduced that the high resistance rate of macrolides in China was related to the distribution of *emm* types.

The distribution of *emm* genotypes of GAS varied according to the countries, regions, and periods. *emm*1 is the most popular type in Germany, consistent with that in the USA, Australia, and Japan; the prevalent types were *emm1* (31.8%), *emm28* (15.4%), and *emm 89* (14.5%) [14, 32]. Presently, the most popular *emm* types in China are *emm12* and *emm1*. In 2011, two patients with scarlet fever died in Hong Kong; the GAS pathogens were *emm1* and *emm12* [33]. In Chaoyang district, Beijing, in 2011, the main GAS epidemic strain of scarlet fever in children was *emm12.0* [34]. In our study, 297 GAS isolates were recovered from patients with scarlet fever at the Children’s Hospital from 2016 to 2017. Nine *emm* types, including 28 subtypes, were identified, of which, *emm12* (65.32%, 194/297) and *emm1* (27.61%, 82/297) were the most prevalent *emm* types (Table 4). In a previous study, eight *emm* types were identified in 155 isolates of GAS recovered from the pharynx of children with scarlet fever, pharyngeal tonsillitis, as well as healthy carrier in Beijing. *emm1.0* and *emm12.0* were the main types of scarlet fever and pharyngeal tonsillitis. *stg485*, *emm18.0*, *emm1.0*, and *emm12.0* were the main types of healthy carrier [35]. From 2009 to 2016, the main *emm* types of GAS strains were *emm12* (42.9–62.2%) and *emm1* (30.7–35.0%) [25, 36]. Interestingly, the proportion of *emm12* and *emm1* in this study was similar to that reported previously. These results showed that the *emm* genotypes of GAS isolates changed significantly in recent years as compared to those identified in the 1990s. The most common *emm* genotypes in 1993–1994 were *emm3.1*, *emm1.0*, *emm4.0*, emm12.0, *st1815.0*, *emm6.0*, and *emm18.0* [37]. You Y collected 2484 strains of GAS during 2011–2018 and found that the prevalent *emm* types of GAS causing scarlet fever shifted for 8 years in Beijing since 2012, the frequency of *emm12 S pyogenes* started to decline from 2011, whereas *emm1* started to increase and then exceeded *emm12* in 2013 and 2014. Since 2015, *emm12* exceeded *emm1* and became the main type again. Notably, numbers of nonpredominant types *emm128* increased substantially in 2017 and of *emm3* in 2018 [38].

The main types of GAS in China are different from those in other countries around the world. A total of 35 *emm* types in 1282 isolates from GAS infection in children in Greece from 2007 to 2013, included *emm1* (16.7%), *emm12* (13.6%), *emm77* (10.9%), *emm6* (6.8%), and *emm89* (6.6%) [1]. Among 1122 invasive isolates from Finland during 2008–2013, 72 *emm* types were identified, of which *emm28* (26%), *emm89* (12%), and *emm1* (12%) were the most common types [39]. The main *emm* types of iGAS strains in Portugal from 2010 to 2015 were *emm1* (28%), *emm89* (11%), *emm3* (9%), *emm12* (8%), and *emm6* (7%) [31]. Furthermore, the isolates of *emm60.1* and *emm63.0* genotypes were prevalent in the children from the villages of Guizhou Province in China, which led to the outbreak of acute glomerulonephritis in 2005 [40]. In 2012, many people suddenly had a fever, sore throat and/or fatigue, headache, and other similar symptoms within 24 h in Beijing. The isolated GAS strain had the same genotype (*emm89*), which was first discovered to cause tonsillar pharyngitis in Beijing, China [41]. *emm89*was also identified in this study. Between January 2016 and May 2017, a rare outbreak of GAS, caused by *emm66.0*, occurred in England and Welsh [42]. The local outbreak of GAS infection is related to the shift of *emm* types. Moreover, different *emm* types carry different resistance genes, which leads to the difference of erythromycin resistance rate. In erythromycin-resistance isolates in Brazil the *ermB* gene was predominant, followed by the *ermA* gene. Thirty-two *emm* types and subtypes were found, but five (*emm1, emm4, emm12, emm22, emm81*) were detected in 48% of the isolates [28]. These results were different from that in China [21]. Therefore, continuous monitoring of streptococcal infection is required.

GAS superantigens, except *speG*, *speJ*, and *smeZ* encoded by chromosome, *speA*, *speC*, *speH*, *speI*, *speK*, *speL*, *speM*, and *ssa* are encoded by phage, which is the main driving force for pathogenic strains to obtain pathogenic factors through transfer. The transfer and mutation of genes can produce highly pathogenic GAS strains, which affect the epidemic situation of the GAS disease, resulting in different distributions of the *S. pyogenes* superantigen gene spectrum in different periods and geographical areas. A study from Portugal showed that *smeZ* (96.0%) and *speG* (86.9%) were common in GAS, followed by *speC*, *ssa*, *speJ*, *speA*, *speK*, and *speI* [43]. A multicenter study in China has proved that 31.1% of the GAS isolates harbored *speA*, while 58.6% harbored *speC* [17]. *emm1* and *emm12* were consistently the most prevalent types of GAS isolates from pediatric patients during 1993–1994 and 2005–2006. Isolates carrying six or more superantigen genes increased from 46.53% in 1993–1994 to 78.39% in 2005–2006. The level of *ssa*, *speH*, and *speJ* genes increased, while that of *speA* decreased. The gene profiles of superantigen were associated with the *emm* type, but strains of the same *emm* type occasionally carry different superantigen genes in the two periods. Intriguingly, no significant difference was detected in the distribution of *emm* types and *SAg* gene profile between isolates from different diseases [37]. In this study, 11 superantigens were detected in GAS isolates, and *speC*, *speG*, and *smeZ* were the most common superantigens. *emm1* harbored *speA*, *speC*, *speG*, *speJ*, *speM*, *ssa*, and *smeZ*, but the content of *speI*, *speK*, *speL* was less. *emm12* type tended to contain *speC*, *speG*, *speH*, *speI*, *speM*, *ssa*, and *smeZ*, with little or no *speJ*, *speK*, and *speL*. A study from Germany showed that the most common superantigen genes in GAS were *speG* (92.1%), *speJ* (50.9%), and *speC* (42.0%). Simultaneously, it was observed that *emm* types or superantigen genes had significant correlation with clinical complications [14]. In an outbreak of GAS infection caused by a rare *emm58* type in a multiple trauma treatment center, it was found that the strain was macrolide and tetracycline resistant and produced the Streptococcal exotoxins *speB* (a streptococcal cysteine protease)*, speC, speG, smeZ and speF* (now considered to be a DNase) [44]. From 2009 to 2016, all isolates from infected patients in 10 general tertiary hospitals in 7 provinces (cities) of China, whether invasive or no-invasive, harbored genes for the protease *speB* and the pore-forming toxin *slo*. The other virulence genes, *smeZ*, *speF*, and *speC* accounted for 96.4, 91.4, and 87.1% of collected isolates, respectively. All strains were sensitive to penicillin, ampicillin, cefotaxime, and vancomycin, whereas the resistance rates to erythromycin, clindamycin, and tetracycline were 93.5, 94.2, and 86.4% respectively, which indicated high genotype diversity and high prevalence of macrolide resistance of *S. pyogenes* in clinical isolates circulating in China [25]. In the previous studies on children, 30.5 and 57.2% of GAS isolates harbored superantigen genes *speA* and *speC*, respectively. 88.8% of *emm1.0* genotype strains contained the *speA* gene, while 69.6% of the *emm12.0* genotype strains contained the *speA* gene [17]. In Taiwan, isolates with *emm1.0*, *emm4.0*, and *emm12.0* genotype were the main causes of non-invasive diseases. A few isolates with *emm1.0* genotype harbored *speC* and *speH* genes, while a few isolates with *emm12.0* genotype harbored *speJ* and *smeZ* genes [45]. In Spain, the isolates of *s.pyogenes* with *emm1.0* genotype, associated with pharyngitis, carried *speA, speG* and *speJ* genes, but did not carry *speC, speH, speI* or *ssa* genes [46]. All the above studies showed that the distribution of *emm* genotypes and superantigen gene profiles were time and region dependent.

## Conclusions

The classification of pathogenic microorganisms is essential for epidemiological research. In this study, all GAS isolates from the Children’s Hospital were sensitive to penicillin, ceftriaxone, and vancomycin, and highly resistant to erythromycin and clindamycin. Nevertheless, penicillin can still be used as the first option for the treatment of streptococcal infections. *Emm* gene typing study shows that *emm12* and *emm1* are the most prevalent genetypes. *ermB* gene is a key role of Streptococcus in the resistance to macrolides, and *speC*, *speG*, and *smeZ* are the most common superantigens of GAS. The long-term monitoring of the *emm* types and superantigens of GAS is crucial for understanding the variations of GAS M protein, the generation of new bacterial type, the prediction of epidemic trends and the preparation of vaccine.

## Data Availability

The datasets generated and/or analyzed during the current study are available from the corresponding author on reasonable request.

## Abbreviations

GAS: Group A streptococcus
*emm*: Encoding mature M Protein gene
CLSI: Clinical and Laboratory Standards Institute
MIC: Minimum inhibitory concentration
CDC: Center for Disease Control and Prevention
PCR: Polymerase chain reaction
